# Fructose Consumption in the Development of Obesity and the Effects of Different Protocols of Physical Exercise on the Hepatic Metabolism

**DOI:** 10.3390/nu9040405

**Published:** 2017-04-20

**Authors:** Rodrigo Martins Pereira, José Diego Botezelli, Kellen Cristina da Cruz Rodrigues, Rania A. Mekary, Dennys Esper Cintra, José Rodrigo Pauli, Adelino Sanchez Ramos da Silva, Eduardo Rochete Ropelle, Leandro Pereira de Moura

**Affiliations:** 1 Laboratory of Molecular Biology of Exercise (LaBMEx), School of Applied Science, University of Campinas, 13484-350 Limeira, Brazil; rodrigo_mpereira@hotmail.com (R.M.P.); jdbotezelli@yahoo.com.br (J.D.B.); kellen.rodrigues.nut@gmail.com (K.C.d.C.R.); jose.pauli@fca.unicamp.br (J.R.P.); eduardo.ropelle@fca.unicamp.br (E.R.R.); 2 Department of Nutrition, Harvard T. Chan School of Public Health, Boston, MA 02115, USA; rmekary@hsph.harvard.edu; 3 Department of Social and Administrative Sciences, MCPHS University, Boston, MA 02115, USA; 4 Laboratory of Nutritional Genomics (LABGeN), School of Applied Science, University of Campinas, 13484-350 Limeira, Brazil; dennys.cintra@fca.unicamp.br; 5 School of Physical Education and Sport of Ribeirão Preto, University of São Paulo, 14049-900 Ribeirão Preto, Brazil; adelinosanchez@usp.br

**Keywords:** fructose, obesity, liver, aerobic exercise, strength exercise, combined exercise

## Abstract

Fructose consumption has been growing exponentially and, concomitant with this, the increase in the incidence of obesity and associated complications has followed the same behavior. Studies indicate that fructose may be a carbohydrate with greater obesogenic potential than other sugars. In this context, the liver seems to be a key organ for understanding the deleterious health effects promoted by fructose consumption. Fructose promotes complications in glucose metabolism, accumulation of triacylglycerol in the hepatocytes, and alterations in the lipid profile, which, associated with an inflammatory response and alterations in the redox state, will imply a systemic picture of insulin resistance. However, physical exercise has been indicated for the treatment of several chronic diseases. In this review, we show how each exercise protocol (aerobic, strength, or a combination of both) promote improvements in the obesogenic state created by fructose consumption as an improvement in the serum and liver lipid profile (high-density lipoprotein (HDL) increase and decrease triglyceride (TG) and low-density lipoprotein (LDL) levels) and a reduction of markers of inflammation caused by an excess of fructose. Therefore, it is concluded that the practice of aerobic physical exercise, strength training, or a combination of both is essential for attenuating the complications developed by the consumption of fructose.

## 1. New Story/Old Enemy

The high consumption of sugary beverages rich in fructose is directly related to the development of obesity and its consequences, such as metabolic syndrome [1,2,3]. Concomitant with the increased incidence and prevalence of obesity and metabolic syndrome, the consumption of fructose has increased around 30% in the last 40 years [4]. More specifically, because fructose is less able to promote satiety and is more palatable, it will stimulate a higher consumption of food [4], and alter the metabolism of lipids and carbohydrates, thereby favoring the synthesis and accumulation of fat [5]. The accumulation of adipose tissue has come to be considered a global public health problem. The hypertrophy of this tissue generates harmful effects on the organism through the secretion of various types of adipokines, and for this reason obesity happens to be considered one of the major risk factors for the development of metabolic syndrome and, consequently, is listed as one of the most serious problems in relation to quality of life [6]. According to epidemiological data, it is expected that by 2025, approximately 18% of men and 21% of women worldwide will be considered obese [7]. With that in mind, because fructose consumption is strongly associated with the development of obesity, studies aimed at evaluating its role in the development of obesity are of paramount importance for a better understanding of the development process of obesity.

A recent meta-analysis found that consumption of fructose-rich beverages leads to increased body weight gain, elevated systolic blood pressure, hyperglycaemia, hyperinsulinaemia, and increased serum triglyceride (TG) concentrations [8]. On the other hand, it was demonstrated that the replacement of fructose by glucose in beverages for 4 weeks resulted in an improvement in insulin sensitivity in adipose tissue in young subjects diagnosed with non-alcoholic fatty liver disease (NAFLD) [9]. The harmful effects of fructose can also be found from the first months of life. Newborn babies, who were breastfed by mothers who had ingested this sugar during pregnancy or lactation, presented metabolic alterations that may last throughout life. Zheng and collaborators [10] showed that children of mothers who consumed fructose had increased body weight, food intake, and circulating levels of leptin, and decreased insulin sensitivity. Later Hu and collaborators [11] demonstrated that each glass or can of fructose-enriched beverage ingested daily by a child increases by up to six times the probability of that child becoming obese during adulthood [12], thereby listing fructose as an important sugar in the genesis of obesity. Therefore, this article aimed to review the current literature to clarify how high fructose consumption may lead to metabolic diseases in rodents and humans, and how different types of physical exercise might be important to attenuate those complications.

## 2. Methodology

The present study is characterized as a narrative literature review. The articles used in this review were searched in the electronic database PubMed (Medline), using the following descriptors and their combinations: fructose, high fructose, obesity, aerobic exercise, endurance exercise, aerobic training, endurance training, strength exercise, resistance exercise, strength training, resistance training, combined exercise, concurrent exercise, combined training, and concurrent exercise. Moreover, to select the articles related to the aim of this review, the title and summary of searched publications were read [13]. When the articles matched to the objectives of this review, they were fully read. The research was conducted in November 2016, and even though the authors did not set limits for the year of publication, the most recent articles about this issue were preferred. Furthermore, only full text articles were used in this review.

## 3. High Fructose Intake and Its Consequences on Metabolic Health

### 3.1. Animal Evidence

As previously discussed, the rates of obesity and fructose consumption are following the same pattern; thus, several studies were conducted to understand how fructose consumption was related to weight gain. In an experiment in which rodents were fed fructose or sucrose during 8 weeks, the authors observed that even though there were no differences between the total caloric intake, animals fed fructose gained more weight than those fed sucrose [14]. In the same study, when the intervention period lasted 6 months, male and female rodents fed fructose had an accumulation of adipose tissue mainly in the abdominal area and an increase of serum TG levels.

Moreover, several studies corroborated with the evidence that high fructose consumption might lead to accumulation of adipose tissue, systemic inflammation, obesity, oxidative stress, and consequently insulin resistance in different tissues [15,16,17,18]. Furthermore, mice who received fructose in their water had an elevation of serum proteins with proinflammatory activity, such as interleukin 1β, interleukin-6, and tumor necrosis factor alpha (TNF-α) [15]. Rodrigues and colleagues [17] demonstrated that the replacement of sucrose for fructose in one meal was enough to establish inflammation in liver and in adipose tissue. In addition, one hour after intervention, TNF-α levels of those fructose fed animals were higher than the sucrose fed animals, and this raise remained for at least 4 h. A similar behavior was observed with interleukin-6 in adipose tissue. Another study demonstrated the antioxidant system impairment of animals fed a diet composed by 60% of fructose, where those animals presented increased malondialdehyde levels [16]. Moreover, the authors further demonstrated that both fasting and homeostasis model assessment-estimated insulin resistance (HOMA-IR) were elevated after 8 weeks of treatment with high fructose diet, indicating severe insulin resistance [16].

Furthermore, high fructose consumption may contribute to the development of obesity and metabolic complications, since it affects the central nervous system and might disturb hunger and satiety control. Rodents hydrated with fructose solution showed higher levels of ghrelin than animals hydrated with solutions of sucrose or glucose [19]. Moreover, the anorexigenic neuropeptides expression was reduced in all groups treated with carbohydrates, but only the group that received fructose solution presented an elevation in cannabinoid 1 (CB1) receptor messenger RNA (mRNA) levels. In addition, Huang and colleagues [20] observed that after an 8-week intervention, the leptin levels were approximately 100% higher in animals fed high fructose diet than those fed chow diet.

On the other hand, liver seems to be a key organ in the development of metabolic complications related to fructose-rich diets [21,22,23,24]. Wistar rats were fed diets containing 65% of the calories from fructose, and the accumulation of fat and collagen in the liver tissue was as evident as observed in animals fed a diet rich in saturated fat, promoting apoptosis in this tissue and activation of proteins related to endoplasmic reticulum stress and inflammatory process [21]. Recently, an important study with primates showed that diets rich in fructose were able to induce a hepatic steatosis stage, with lipid droplet size positively correlated to time of exposure to the diet [24]. Moreover, they observed that high fructose consumption might induce liver damage even without excessive intake of fat or calories.

### 3.2. Human Evidence

Given the robustness of the evidence presented by basic research on the deleterious effects of high fructose consumption on metabolic parameters, it is expected that excessive fructose intake might lead to metabolic complications in humans. In 2010, Goran and colleagues [25] demonstrated that countries which used fructose-rich sweeteners had a higher incidence of diabetes, even though the incidence of obesity and the total amount of sugars ingested did not increase. Recently, Lin and colleagues [26] carried out a study including 1454 adolescents, in which they observed that high fructose consumption was related to higher levels of fasting insulin, serum uric acid, and central adiposity.

Furthermore, some studies in humans have also shown an increase in circulating TG levels due to high fructose consumption [27,28]. Silbernagel and colleagues demonstrated that after consuming 150 g of fructose daily for 4 weeks, subjects presented an elevation of 350 mg/L of serum TG levels, while for participants who consumed the same amount of glucose, their TG levels remained unchanged [27]. In addition, Abdel-Sayed and colleagues [28] observed that TG levels may increase earlier, with only 7 days of fructose supplementation. These authors also demonstrated that high fructose diet increased lactate production and hampered mobilization and lipid oxidation. Similar results were found by Lê and colleagues [29] in a study where healthy subjects increased very low density lipoprotein (VLDL) level after one week consuming fructose solutions thrice daily. Moreover, leptin levels of these subjects were also increased after one week of intervention, reaching a 48% raise after four weeks of experimentation. On the other hand, healthy women did not present changes in any parameters related to hunger and satiety control, such as insulin, leptin, and ghrelin levels after a fructose-rich meal [30]. Therefore, more studies are needed to better understand how variables such as dietary exposure time, fructose concentration, and number of daily meals may influence the level of hormones related to hunger and satiety control in humans.

Moreover, liver is one of the main targets of the harmful effects of high fructose consumption. Schwarz and colleagues [31], using magnetic resonance spectroscopy, observed that 9 days of fructose-rich diet was enough to raise liver lipids accumulation with a significant increase in postprandial de novo lipogenesis and complications in the control of hepatic glucose production. Sobrecases and colleagues [32] found similar results in their study with healthy men who received isocaloric solutions of fructose or saturated fat for 7 days. At the end of the intervention period, the intrahepatocellular lipids accumulation was similar between the groups. On the other hand, only the fructose solution group increased VLDL levels. Thus, the authors concluded that a short-term intervention with fructose-rich solution might promote fat liver accumulation and contribute to the development of insulin resistance in this tissue.

## 4. The History of Fructose Consumption

Sucrose has been widely used since the Middle Ages as a dietary component. It was originally derived from sugar cane in countries such as New Guinea and the Indian subcontinent, from where it was transported to Europe where it was consumed only by royalty and the most fortunate. In the fifteenth century, the countries of the Iberian Peninsula began to increase the planting of sugar cane and sugar production. However, only after the 1500s, with the discovery of the Americas and the use of slave labor, did cane planting and sugar export begin to expand. Consequently, with the increase in its production, sugar began to be consumed by the whole population, becoming widely used for the production of sweets during the eighteenth century, so that the average consumption per capita of sugar in England jumped from 1.8 km in the year 1700 to 8.1 km in 1800 [4]. Finally, it was only in the 1960s that fructose was included as a sweetener in the diet with the production of “sweet corn-based syrups” known as “high-fructose corn syrups” (HFCSs) [33].

The inclusion of HFCS as a sweetener brought benefits such as longer shelf life and lower cost [4]. Thus, the creation of HFCS-42 in 1967 and HFCS-55 in 1977 (HFCS-42 consisted of 42% and HFCS-55 consisted of 55% fructose, respectively) promoted new opportunities for the sweetener and beverage industries. Since then, consumption of sucrose and HFCS has grown exponentially. In the 1970s, syrup accounted for less than 1% of the calories ingested through caloric sweeteners in the United States, reaching a rate of 42% in the 2000s and it is currently found in most foods containing caloric sweeteners [34]. While efforts to combat the development and treatment of obesity are rising, food production containing fructose, sucrose, or HCFs is increasing quickly. Currently, an American individual consumes, on average, 72 g/day of sugar, corresponding to approximately 275 kcal/day [35]. Over the years, syrups rich in fructose have been produced from a variety of other raw materials, such as sugar cane, tapioca, rice, wheat, manioc, and beet [33]. This led several research groups to identify the intake of this nutrient as the main engine of the current obesity pandemic [36,37].

## 5. Sweet Poison

Found in several processed foods, fructose is usually either bonded to glucose molecules (sucrose) or not (HFCS). After ingestion, the fructose molecules pass through the digestive tract and reach the small intestine where they are rapidly absorbed by the intestinal epithelium through the glucose transporters (GLUT5) [38] and then released into the bloodstream. In the bloodstream, this nutrient is absorbed by different tissues, but mainly by the liver, which has high amounts of glucose transporter 2 (GLUT2) [38]. On the other hand, virtually no fructose is absorbed by pancreatic beta cells, because they lack expressive amounts of GLUT2 and GLUT5 transporters [39]. This characteristic is extremely important for understanding the pathogenesis of obesity. While glucose triggers the release of insulin by pancreatic beta cells, fructose is not able to do so [35]. In addition, this nutrient also appears to not stimulate leptin release and does not suppress the release of ghrelin in starvation [35,40]. These three peptide hormones play a fundamental role in the control of food intake and basal energy expenditure, acting both in the central nervous system and peripheral tissues [41,42]. While ghrelin increases the forkhead box protein 01 (FoxO1) binding to deoxyribonucleic acid (DNA), both insulin and leptin phosphorylate FoxO1 release it from DNA, thereby reducing the hunger signal and hepatic gluconeogenesis and contributing to increased energy expenditure [43,44]. Animal studies have shown that following the administration of fructose directly into the hypothalamus, rodents showed increased food intake, while glucose injection had the opposite effect [45]. These findings explain in part the increased prevalence of obesity in individuals who consume this nutrient in the form of sugary drinks or industrialized foods [11]. Although the lack of effects on satiety, energy expenditure, and glucose uptake in itself is extremely damaging, fructose also activates extremely harmful signaling pathways in liver tissue cells.

Most cells have reduced GLUT2 content, which leads to a marked transport of this nutrient to the hepatocytes where the presence of these transporters is abundant [38]. Inside the cytoplasm, fructose may provide an energetic substrate for hepatic glucose production (gluconeogenesis) or be rapidly phosphorylated and converted to fructose 1-phosphate (fructose 1-P) by the action of the enzyme fructokinase, which uses the energy of an adenosine triphosphate (ATP) molecule. This conversion decreases energetic availability in the hepatocyte and increases the contents of intracellular adenosine diphosphate (ADP) and adenosine monophosphate (AMP). Elevated levels of ADP and AMP activate mitochondrial energetic pathways, increasing the NAD^+^/NADH (nicotinamide adenine dinucleotide) ratio. An increased NAD^+^/NADH ratio leads to increased activity of Sirtuin-1 (SIRT-1) and phosphoenolpyruvate carboxykinase (PEPCK) [46]. Finally, the strong deacetylation activity of SIRT-1 [25] deacetylates the already known FoxO1 protein, increasing its binding to nuclear DNA and triggering the expression of the protein kinase C (PKC) and peroxisome proliferator-activated receptor-gama coactivator 1 alpha (PGC-1α) genes [47,48]. All this fine mechanism triggered by the simple elevation of fructose in the intracellular environment results in increased rates of hepatic gluconeogenesis and hyperglycaemia. In addition to the effects on the control of glycaemic homeostasis, increased AMP concentration triggered by fructose activates the AMP deaminase enzyme, starting the hypoxanthine pathways, which increases the inflammatory process and produces uric acid. Uric acid is a potent inhibitor of the nitric oxide synthase enzyme that acts on the production of nitric oxide by the conversion of arginine to citrulline [49]. Nitric oxide plays a key role in endothelial relaxation leading to vasodilation through increased lumen diameter of the arteries. As a result, fructose, through increased levels of uric acid, may prevent the proper functioning of blood vessel gauge control pathways and contribute to elevated systemic blood pressure [50]. All these mechanisms initially triggered by the transport of fructose into the hepatocyte are extremely relevant and contribute to the development of diseases such as hyperglycaemia, gout, endothelial inflammation, and arterial hypertension [2,51].

Raising the levels of fructose-1P inside cells activates other important energy pathways. The 1P form of this nutrient activates peroxisome proliferator-activated receptor-gama coactivator 1 beta (PGC1-β) protein, which in turn increases the expression of the sterol regulatory element-binding protein 1c (SREBP1c). SREBP1c initiates the transcription of fatty acyl-coA synthase (FAS) and acetyl-CoA carboxylase (ACC) proteins [52]. All these mechanisms prepare the cell for an increase in fatty acid synthesis using the carbon chains supplied by intracellular fructose. The fructose-1P is converted into glyceraldehyde and dihydroxyetonephosphate, two intermediates of glycolysis. This process, called “fructolysis”, requires the activity of the enzyme fructose-1P aldalose [53]. Glyceraldehyde provides carbon chains for the production of pyruvate, which goes to the mitochondria, where it is reduced to Acetyl-CoA. In the mitochondrial matrix, Acetyl-Coa is converted to citrate through the Krebs cycle and then migrates from the mitochondria to cytoplasm, where it will be converted into malonyl-CoA by the enzyme ACC. The excess of malonyl-coA in cytoplasm inhibits the activity of the protein carnitine palmitoyl transferase 1 (CPT-1), thereby blocking the transport of lipids to the mitochondria, and stopping the β-oxidation [54]. Malonyl-coA will be converted to acyl-coA by the enzyme FAS (transcribed by increased activity of SREBP1c). This fatty acid now has three different targets in the cell. Part of the acyl-coA produces triglyceride molecules that accumulate in the hepatocyte, leading to non-alcoholic fatty liver disease. Another amount binds to apolipoprotein (ApoB) to produce VLDL, or simply diffuses in the form of free fatty acids into the bloodstream, triggering hypercholesterolaemia and dyslipidaemia [1,53]. The excessive influx of lipids can now reach the white adipose tissue, generating white adipose tissue (WAT) hypertrophy of it; the skeletal muscle, where it triggers insulin resistance [55]; or the pancreas, inhibiting the production and secretion of insulin. Finally, high levels of acyl-coA can be converted to diacylglycerol (DAG) by diacylglycerol acyltransferase [56]. DAG activates the protein kinase C epsilon (PKCε), which, in turn, activates the protein c-jun-*N* terminal kinase-1 (JNK1) [57]. This protein leads to hepatic insulin resistance through the phosphorylation of IRS-1 on Serine307 residue (IRS-1Ser307). This mechanism of hepatic insulin resistance perpetuates the sign of hepatic gluconeogenesis, leading to a marked increase in blood glucose and contributing to weight gain [34,58]. In 2000, Ueno and collaborators (2000) observed that insulin signaling was reduced by nearly 72% in the hepatic tissue of rodents exposed to a fructose-rich diet for 28 days [59]. In addition to inducing hepatic insulin resistance, activation of JNK-1 activates transcription factor 1 (AP-1). AP-1 transcribes inflammatory genes and activates the synthesis of inflammatory cytokines by the hepatocyte. Once released into the extracellular environment, these cytokines will bind to cytokine receptors in Kupffer cells. These cells perpetuate the inflammatory signal and can further overwhelm the hepatocyte through the release of reactive oxygen species and cytokines [60]. Both alcohol consumption and fructose consumption activate the formation of reactive oxygen species and increase the expression of inflammatory proteins in the hepatocyte, contributing to tissue damage and inflammation through this tricky process [36,61]. In addition, fructose can exist in two different stereoisomeric forms: one linear (ketone form) and the other in the form of a furanosidic ring (fructofuranose). The ratio of both forms depends on the pH and temperature of the medium. In the bloodstream, most of the fructose is in the linear form, with the ketone group exposed and susceptible to fructosylation reactions. In fact, fructose fructosylation releases large amounts of superoxide anion, leading to the disproportionate formation of reactive oxygen species [62]. The expressive increases of reactive oxygen species (ROS) leads the system to increase the antioxidant response by abruptly raising the expression of reducing proteins [63]. This response may be compromised in children and adults with micronutrient deficiency, leading to cellular and tissue damage [64]. Even under ideal micronutritional conditions, long-term administration of fructose can result in the failure of the antioxidant system [65,66]. In addition, this imbalance in the redox state and increased cell damage also lead to increases in JNK phosphorylation and activation of the AP-1 transcription factor. This pro-inflammatory additive perpetuates the insulin resistance and hepatic lipogenesis [67]. Finally, the excessive consumption of fructose produces a link between white adipose tissue and the liver [68]. Hypertrophy of white adipose tissue triggers an increased release of inflammatory cytokines by the adipocyte. Among these cytokines is the tumor necrosis factor family. In a study published by our group in 2016, we showed that levels of TNF-α in animals fed a high fructose diet were practically doubled [69]. These cytokines bind to specific cellular receptors and activate the cascade to perpetuate the inflammatory signal and insulin resistance. In the liver, these cytokines may increase the expression of another family of inflammatory receptors, the toll-like receptors (TLRs) [70], leading to overlapping systems repeating an already deregulated process of inflammatory feedback. In the skeletal muscle, the presence of these cytokines can trigger insulin resistance [69]. In the central nervous system, the presence of inflammatory cytokines prevents the efficient signaling of leptin and insulin by inhibiting the effect of these peptides on food consumption, energy expenditure, and central control of hepatic gluconeogenesis through reduced FoxO1 phosphorylation [44].

**Figure 1:**
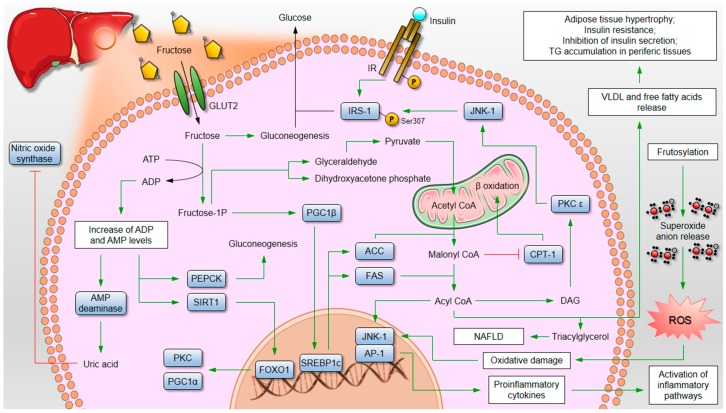
Role of fructose on metabolic diseases. Fructose reduces the phosphate biodisponibility, leading to acid uric production and nitric oxide synthase inhibition contributing to hypertension. Reduced phosphate biodisponibility also activates SIRT-Dependent deacetylase of FoxO1 contributing to gluconeogenesis and hyperglycemia. Fructose-1P upregulates PGC-1β expression by promoting lipogenesis through SREBP1c activation. The same nutrient provides carbon chains for the synthesis of triglycerides, diacilglycerides, and VLDL cholesterol contributing to hypertriglyceridemia, hepatic insulin resistance, and dyslipidemia. Sub products of fructose target other tissues, leading to systemic insulin resistance and inflammation. Finally, ROS generated by fructosylation increases oxidative damage and stress response in the inner of cell, leading to DNA damage and proinflammatory cytokines production. ACC: acetyil-coA carboxylase; ACC: Acetyl-CoA Carboxylase; ADP: Adenosine Diphosphate; AP-1: Activator Protein-1; ATP: Adenosine Triphosphate; CPT-1: Carnitine Palmitoyl Transferase 1; DAG: Diacylglycerol; FAS: Fatty Acyl-CoA Synthase; FoxO1: Forkhead box protein 01; Fructose-1P: Fructose 1-Phosphate; GLUT2: Glucose Transporter 2; IR: Insulin Receptor; IRS-1: Insulin Receptor Substrate 1; JNK-1: C-Jun-*N* terminal kinase-1; NAFLD: Non-Alcoholic Fat Liver Disease; PEPCK: Phosphoenolpyruvate Carboxykinase; PGC-1α: Peroxisome Proliferator-Activated Receptor-Gama Coactivator 1 Alpha; PGC-1β: Peroxisome Proliferator-Activated Receptor-Gama Coactivator 1 Beta; PKC: Protein Kinase C; ROS: Reactive Oxygen Species; SIRT-1: Sirtuin-1; SREBP1c: Sterol Regulatory Element-Binding Protein 1c; TG: Triglycerides; VLDL: Very Low Density Lipoprotein.

All cascades exposed so far perpetuate the harmful signal of fructose metabolism in the hepatocyte by activating a vicious cycle that can only be stopped by replacing this nutrient with another in the diet [71,72,73], as shown in Figure 1.

## 6. How to Deal with the Enemy

Several studies have demonstrated evidence that fructose is a nutrient with great obesogenic potential, associated with several metabolic complications and the promotion of de novo lipogenesis [1,74].

Together with obesogenic stimuli from nutritional factors, epidemiological studies also show that the genesis of obesity in contemporary society is also linked to the progressive decrease in the time available for the practice of physical activities by the global population [75]. Thus, it is strongly proposed that exercise is an important tool for combating weight gain and its associated complications, acting in the prevention and treatment of the deleterious changes promoted by high consumption of fructose. However, different models of exercise have been proposed for an improvement in metabolic health, such as aerobic exercise, strength training, and the combination of both. Therefore, we will discuss the different models separately.

### 6.1. Fructose Consumption and Its Complications: The Role of Aerobic Exercise

The knowledge that aerobic exercise is capable of promoting improvement in metabolic health is not recent. Studies performed at the beginning of the 20th century already provided information that physical exercise could potentiate the action of insulin, and thus increases the uptake and utilization of glucose [76]. Recently, studies using immunofluorescence staining technique demonstrated that the glucose transporters 4 (GLUT4) are stored in vesicles in the intracellular environment during rest. However, shortly after an exercise session, the GLUT4 are homogeneously redistributed by the plasma membrane, as well as when there is insulin stimulation [77]. One of the main mechanisms proposed for this phenomenon involves the activation of the protein sensitive to AMP intracellular levels, the AMP-activated protein kinase (AMPK), which is considered essential for the control of energy balance [78]. When activated, AMPK promotes the phosphorylation and activation of Akt substrate that weighs 160 kDa, the AS160. This protein, will promote the release of GLUT4, allowing the transporter to go to the cell membrane via independent mechanisms of insulin action [79,80].

Furthermore, Matos and colleagues [81] demonstrated that, when stimulated by insulin, obese animals that were submitted to an aerobic exercise session showed an insulin signaling pathway activation similar to the control group, while the sedentary obese animals showed a consistent reduction in this activation. It is suggested that this effect may be caused by the fact that aerobic exercise reduces the levels and activity of pro-inflammatory proteins [82,83], and protein-tyrosine phosphatase 1B (PTP-1B), thereby reducing the insulin resistance state [82]. Therefore, aerobic exercise increases both insulin action in skeletal muscle and glucose uptake by mechanisms that are independent of the action of this hormone. Thus, we can infer that aerobic exercise provides an agonist action of insulin in the skeletal muscle. However, the improvement of metabolic process promoted by this type of exercise is not only limited to skeletal muscle [81,82], but it also extends to other key tissues such as the liver [84,85], hypothalamus [86,87], and adipose tissue [85].

In the hepatic tissue, our group demonstrated that with only one exercise session, it is possible to reduce the levels of PTP-1B [84], a protein which is able to down-regulate insulin signal transduction [88]. The same study also found decreased levels of proteins involved in gluconeogenesis, such as PEPCK and glucose-6-phosphatase (G6Pase). Aerobic exercise also proved to be able to reduce the phosphorylation of proteins kinase RNA-like endoplasmic reticulum kinase (PERK) and eukaryotic initiation factor 2-α (eIF2α) [85], which are regarded as the greatest stress markers of endoplasmic reticulum [89]. Thus, the phosphorylation of insulin receptor tyrosine and their substrates and the activation of protein kinase B (Akt) increased, while also decreasing the inflammation in this tissue.

In the central nervous system, specifically in the hypothalamus, aerobic exercise seems to influence the control of hunger and satiety. Ropelle and colleagues [86] observed that the energy intake of animals treated with a hyperlipid diet was higher than that of animals treated with commercial feed. The authors also showed that when these same animals were submitted to an aerobic exercise session, including running and swimming, the energy intake of the obese group was equal to the control group within 12 h following the completion of the exercise. In addition, even though there was no reduction in body weight and adipose tissue, mRNA levels of pro-opiomelanocortin (POMC) were increased and the levels of neuropeptide-Y (NPY) were decreased in the hypothalamus of these animals. Rodrigues and co-authors [87] demonstrated that aerobic exercise reduced the phosphorylation and translocation of FoxO1 into the nucleus, thus inhibiting the transcription of orexigenic neuropeptides. Moreover, the protein content and activity of a mammalian homolog of Drosophila tribbles 3 (TRB3)—which may be associated with AKT and down-regulation of insulin signaling in the hypothalamus—was decreased.

Adipose tissue is also the target of molecular changes promoted by aerobic exercise. Besides reducing the amount of fat in different regions [90,91], aerobic training is also able to reduce hypertrophy of adipocytes in obese animals [91], which is essential to improve the systemic inflammatory status promoted by obesity. Notably, when hypertrophied, adipose tissue is responsible for the secretion of a series of proteins with pro-inflammatory activity [6]. Consequently, pro-inflammatory pathways such as JNK and I-kappa-B-alpha (IκBα) are less activated [85].

Once the insulin activity in several tissues that are responsible for the metabolic control has been enhanced, the serum levels of pro-inflammatory proteins [85,87], and fasting glucose [81,92] are reduced. After 8 weeks of endurance training, da Luz and colleagues [85] observed a decrease in serum levels of TNF-α, demonstrating that there was a systemic decrease in the inflammation of obese animals. Likewise, aerobic exercise also reduces oxidative stress and increases antioxidant capacity.

Aerobic exercise is also proposed as a strong strategy for the prevention and treatment of NAFLD. Gauthier and colleagues [93] found that sedentary obese animals had a 72% increase in fat accumulation in the liver, with 48% more lipid vacuoles than the animals treated with a standard diet. However, animals that performed aerobic exercise during the obesity induction period had a reduction in the development of NAFLD. Similar results were found by Shen and colleagues [91], in which the amount of fat observed in the liver of obese and exercised animals was similar to that observed in the control animals. These results were still accompanied by a reduction in the gene expression of stearoyl-CoA desaturase-1 (SCD-1), described as a key regulator of lipid metabolism, so that this deletion provides an improvement in the oxidation of fatty acids machinery in the liver [94]. Charbonneau and colleagues [90] also observed that training the obese animals resulted in equating the liver TG levels to those in the control group.

As discussed previously, the lipogenic properties of fructose are associated with large increases in triglyceride levels [95,96,97]. In this context, the practice of exercise, especially aerobic exercise, has been shown to promote important and consistent effects related to the pathogenesis of dyslipidaemia [92,98]. In an important meta-analysis involving 51 studies, Leon and Sanchez [99] found that after 12 weeks or more of aerobic exercise intervention, the subjects had a mean reduction of 3.7% in triglyceride levels, while high-density lipoprotein (HDL) levels were elevated by 4.6% and low-density lipoprotein (LDL) was reduced by 5%, on average.

#### 6.1.1. Animal Evidence

Finally, there is evidence that aerobic exercise is an important tool to combat various metabolic complications induced by high fructose consumption [69,100]. In a study conducted by Stanišić and colleagues [101], Wistar rats received a 10% fructose solution for 8 weeks, and thereafter a significant increase in insulin levels and severe insulin resistance were observed. However, animals that underwent a running exercise on a treadmill during the experiment had such attenuated complications. Farah and colleagues [102], using the same intervention, observed that animals who received a fructose solution presented glucose intolerance and increased adiposity; however, the practice of moderate aerobic exercise was effective to attenuate these complications.

Our research group [69] showed that aerobic exercise, in addition to reducing the activity of inflammatory proteins in the skeletal muscle of animals fed a high fructose diet, also increased levels of interleukin 10, described as a protein with a potent anti-inflammatory potential [103]. Exercised rodents treated with fructose also had a decrease in oxidative stress markers [102] and NAFLD [100], and a lower TG accumulation in liver tissue [69]. Moreover, Farah and colleagues [102] showed that aerobic exercise was able to prevent hemodynamic and autonomic cardiac dysfunction promoted by high fructose diet. On the other hand, Karaca and colleagues [104] demonstrated that positive adaptations promoted by physical exercise such as increased expression of aquaporin 7 (AQP7), an important protein related to metabolic health of cardiac tissue during exercise, were inhibited by fructose-rich diets.

#### 6.1.2. Human Evidence

Similar to animal models, studies in humans have shown that aerobic exercise is an efficient strategy for inhibiting the harmful effects of high fructose consumption. A study involving healthy humans revealed that even after the daily addition of 75 g of fructose in their diet, physical activity was able to reduce insulin levels and maintain unchanged the triglyceride levels of active volunteers, while, on the other hand, the inactive people showed an increase in this parameter [105]. In addition, Egli and colleagues [106] observed that aerobic exercise (moderate intensity) completely eliminated the deleterious effects promoted by 4 days of diet consumption composed by 30% of fructose.

### 6.2. Fructose Consumption and Its Complicatons: The Role of Strength Exercise

Although we are reaching a century of publications on metabolic syndrome related to aerobic exercise, the same does not apply to strength exercise. In 1984, Miller and colleagues [107], in a longitudinal study of healthy subjects, observed the effects of completion of 10 weeks of isotonic exercise with weights. After the training protocol, participants showed no differences in fasting glucose, but the plasma insulin levels were significantly reduced, from 10.85 ± 1.55 μU/mL to 6.79 ± 1.19 μU/mL. During the glucose tolerance test, the area under the insulinemic curve of subjects submitted to resistance training was also lower than that of those who remained sedentary during the same period, thus bringing the first evidence that strength exercise also has the capacity to improve insulin sensitivity. Finally, the authors also observed an increase in the amount of muscle mass in the trained group, and that these gains had a strong negative correlation with insulin levels during glucose tolerance test. A decade later, Treuth and colleagues [108] showed evidence on the relationship of strength training with adiposity control and, consequently, obesity. After 16 weeks of exercise performed three times per week, the authors observed that although the body weight of participants was not changed, the amount of intra-abdominal adipose tissue was significantly reduced [109].

Recent studies have provided us with a better understanding of how strength exercise is able to improve glycemic control. A performed study which used obese animals and submitted them to strength exercise on staircase [110], besides confirming reductions in serum insulin levels, showed that after 8 weeks of training, some proteins from the insulin pathway had increased their action, such as phosphatidylinositol 3-kinase (PI3-K) and Akt. Furthermore, the GLUT4 gene expression was also increased in these animals. Another study published by the same group of researchers [111] showed that serum levels of TNF-α and interleukin-6 were reduced with training, accompanied by an increase of adiponectin [112]. However, obese subjects undergoing strength training showed improvement in insulin sensitivity even without changes in serum levels of proinflammatory cytokines [113].

Several studies have begun to suggest strength exercise as an important strategy for the prevention and treatment of obesity [114,115,116] and in one of these studies Schmitz and colleagues [115] found positive results in body composition of women who performed strength exercise. In the fifteenth week of intervention, there was a reduction of body fat and an increase in muscle mass. In another state, after one year of intervention, a reduction in the amount of intra-abdominal fat was also observed [116].

After a strength exercise session, positive changes on the lipid profile were observed by Lira and colleagues [117]. However, interestingly, different responses were observed according to the intensity of the exercise. After 72 h of the end of the session, the plasma TG levels were reduced only in subjects who trained in the intensities of 50% and 75% of 1 RM, while those who trained at 90% and 100% showed no differences. Also, after an exercise session, the sensitivity to insulin in the hepatic tissue was improved by 8% ± 1% reductions in glucose production rate and 12% ± 5% glycogenolysis [118].

#### 6.2.1. Animal Evidence

Strength exercise is not as common of an intervention in animal models as aerobic exercise. In a review, Seo and colleagues [119] identified several reasons that hindered the use of this intervention in animals. Strength exercise requires animal voluntary performance, and variables such as intensity and time between sets are difficult to control, thus, the results obtained by these studies are often not very expressive and may be misinterpreted. Because of this, only one study involving strength exercise and fructose consumption was found, and it was carried out by our research group [69]. In summary, the strength exercise promoted consistent improvements in the metabolism of rodents fed a high fructose diet. Similar to aerobic exercise, resistance exercise provided lower levels of glucose and insulin during glucose tolerance test. Moreover, strength exercise decreased interleukin-6 (IL-6) and TNF-α levels. Regarding the liver tissue, strength exercise decreased fat accumulation and led to a greater reduction in the levels of nuclear factor-kappa B (NF-κB) and IκB-α, demonstrating that strength exercise can be promising for the reduction of inflammation promoted by a diet rich in fructose.

#### 6.2.2. Human Evidence

As previously discussed, there are fewer publications about strength exercise than aerobic exercise. The only study that investigated the acute effects of strength exercise with high fructose diet in humans was carried out by Wilburn and colleagues [120]. On the first day of intervention, participants performed 14 different strength exercises (4 sets of 10 repetitions each with 90 s rest between sets) during 95 min. After 15 h of the training session, participants were fed a meal containing 0.75 g/kg body weight (BW) of fructose, and several blood samples were taken for the next 360 min. Although insulin and lactate levels did not differ between the trained and sedentary group after the fructose-rich meal, the postprandial TG concentrations were significantly lower in the trained group. Based on this evidence, the authors concluded that strength exercise performed prior to fructose-rich meals might attenuate elevations in serum TG levels; however, further studies are needed to better understand this issue.

### 6.3. Fructose Consumption and Its Complicatons: The Role of Combined Exercise

In the last few decades, the effects of combined physical exercise have been gaining prominence in the scientific community. This exercise protocol consists of endurance and strength exercises performed in the same training session or on alternate days. Initially, the practice of combined exercise did not seem to be an interesting strategy for metabolic health, since in the 1980s, a classic study revealed that when subjects performed strength exercises and subsequently practiced cycling and running, the strength gains, muscle mass, and consequent increase in body weight were compromised [121]. However, the gains in aerobic performance were not different between individuals who performed aerobic training and those who underwent the combined training. A few years later, the results found by Kraemer and colleagues [122] corroborated the above article, where the authors found that subjects who underwent the combined exercise showed a smaller area in the muscle fibers than those who performed only strength exercise. Thus, it was also shown that combined exercise not only compromised the strength gain promoted by strength exercise, but also hypertrophied different muscle fibers.

However, subsequent studies began to demonstrate evidence that challenged the negative influence of combined exercise on strength gain and cross-sectional area on muscle fibers. In their study, McCarthy and colleagues [123] showed that the combined exercise, performed three times a week for 10 weeks, promoted the same increase in flexor and knee extensor muscle area as strength exercise alone, with no difference in neural activation. In a study by De Souza and colleagues [124], although they did not find hypertrophy after the combined training protocol, they did not find differences in the expression of genes related to the control of protein synthesis, suggesting that the control of other training variables, such as session volume, may be related to these different responses found in the different protocols. Finally, it has also been demonstrated in humans that the activation of proteins involved in protein synthesis, such as the mechanistic target of rapamycin (mTOR) and ribosomal protein S6 kinase beta-1 (S6K1), are not compromised. In fact, AMPK activation [125], a protein that has an increased activity caused by the increase in AMP levels provided by aerobic exercise [83,126], was shown to be able to negatively influence the stimulation of protein synthesis by inhibiting the mTOR pathway in rodents [127]. From this, many other studies have been conducted to better understand the changes promoted by this new and promising exercise protocol, in many different contexts, because it is believed that this type of exercise can activate different pathways and promote several improvements. For these reasons, currently, the practice of combined exercise is widely recommended by the American College of Sports and Medicine [128].

Monteiro and colleagues [129] observed positive effects on body composition in obese adolescents after 20 weeks of combined exercise. At the end of the experiment, the participants presented a reduction in body weight and body mass index (BMI). Interestingly, the magnitude in reducing body fat was very close between the two groups who underwent the combined exercise and aerobic exercise only, and this reduction was 3.5% and 3.9% respectively. Moreover, consistent results were also found in the lipid profile, with reductions in circulating levels of TG and VLDL and increases in HDL levels. On the other hand, the waist circumference of these participants was not different.

Medeiros and colleagues [130] also found positive results of combined exercise on body composition in obese subjects, reflecting an improvement in insulin resistance and oxidative stress markers. Interestingly, the authors used two groups with similar exercise protocols, but one group exercised three times a week, while other exercised 5 times a week. Both groups showed a reduction in body weight and BMI. The group that exercised five times per week reduced the amount of fat mass and increased the amount of muscle mass. The group that performed the training protocol three times per week presented reductions in fasting blood glucose levels and the homeostasis model assessment-estimated insulin resistance (HOMA-IR) index. Reductions in protein carbonylation levels were observed in the group that performed three weekly sessions, indicating an improvement in the antioxidant machinery, since this is a process triggered by ROS [131]. The activity of glutathione peroxidase was decreased and that of lipid peroxidation was increased in both groups. It is known that exercise can moderately increase ROS production, and thus provides positive adaptations for the entire antioxidant system [132]. Thus, these results suggest that combined exercise may be an important strategy for combating insulin resistance and oxidative stress associated with obesity, but more studies are needed to fully understand controls of exercise variables, such as exercise intensity and frequency.

Finally, the combined exercise was also able to present improvements in several deleterious contexts promoted by the high consumption of fructose [69]. Our research group revealed that combined exercise, when performed on alternate days (aerobic and strength on separate days), despite not promoting reduction in body weight, was able to provide an increase in glucose tolerance and insulin sensitivity compared to the aerobic protocol. The circulating levels of HDL were increased, and the reduction of hepatic TG accumulation was strongly visible in histological sections, when compared to the sedentary animals that received high doses of fructose. Finally, exercise also provided a lower activation of Nuclear factor-kappa B (NF-κB), thus presenting a decrease in systemic inflammation. There are no studies on humans that have investigated the role of combined exercise to attenuate the deleterious effects of high fructose diet, thus more studies are needed to fill this literature gap.

## 7. Conclusions

**Figure 2:**
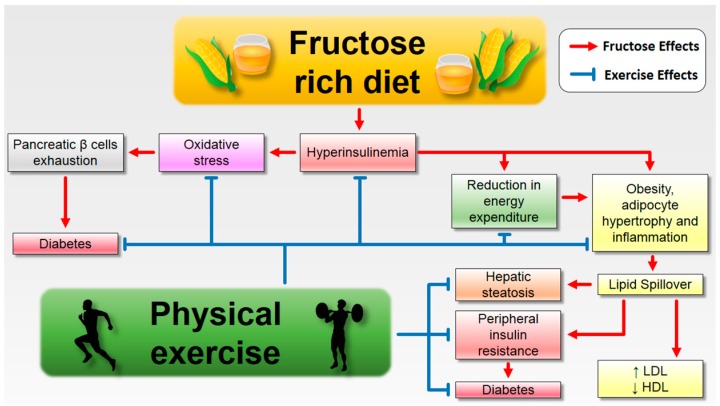
Exercise prevents and treats the deleterious effects of high consumption of fructose. In addition to promoting an increase in energy expenditure, physical exercise consistently attenuates inflammation and oxidative stress related to excessive consumption of fructose, as reflected in positive changes both in lipid profile and fat metabolism. In this way, insulin resistance and hyperinsulinaemia are diminished, collaborating with the prevention and treatment of diseases such as hepatic steatosis and type 2 diabetes. HDL: High-Density Lipoprotein; LDL: Low-Density Lipoprotein.

The consumption of fructose has been increasing along with its harmful effects on the organism. Fructose may trigger changes in the circulating and hepatic lipid profile, favoring the installation of chronic and subclinical inflammation. Regular practice of aerobic physical exercise, strength training, or a combination of both, in turn, has the ability to reverse these parameters, mainly by improving the circulating and tissue lipid profile and reducing inflammation (Figure 2). Therefore, regular practice of physical exercise is an essential tool for attenuating the obesogenic disorders caused by the consumption of fructose.

## Abbreviations

ACC	Acetyl-CoA Carboxylase
ADP	Adenosine Diphosphate
Akt	Protein kinase B
AMP	Adenosine Monophosphate
AMPK	AMP-activated protein kinase
AP-1	Activator Protein-1
AQP7	Aquaporin 7
ATP	Adenosine Triphosphate
BMI	Body mass index
CB1	Cannabinoid 1
CPT-1	Carnitine Palmitoyl Transferase 1
DAG	Diacylglycerol
DNA	Deoxyribonucleic Acid
eIF2α	Eukaryotic initiation factor 2-α
FAS	Fatty Acyl-CoA Synthase
FoxO1	Forkhead box protein 01
Fructose 1-P	Fructose 1-Phosphate
G6Pase	Glucose-6-phosphatase
GLUT2	Glucose Transporter 2
GLUT4	Glucose Transporter 4
GLUT5	Glucose Transporter 5
HDL	High-Density Lipoprotein
HFCS-42	High-Fructose Corn Syrup with 42% of Fructose
HFCS-55	High-Fructose Corn Syrup with 55% of Fructose
HFCS	High-Fructose Corn Syrup
IκBα	I-kappa-B-alpha
IR	Insulin Receptor
IRS-1	Insulin Receptor Substrate 1
JNK 1	C-Jun-N terminal kinase-1
LDL	Low-Density Lipoprotein
mTOR	Mechanistic target of rapamycin
NAD^+^/NADH	Nicotinamide Adenine Dinucleotide
NAFLD	Non-Alcoholic Fat Liver Disease
NF-κB	Nuclear factor-kappa B
NPY	Neuropeptide-Y
PEPCK	Phosphoenolpyruvate Carboxykinase
PERK	Protein kinase RNA-like endoplasmic reticulum kinase
PGC-1α	Peroxisome Proliferator-Activated Receptor-Gama Coactivator 1 Alpha
PGC-1β	Peroxisome Proliferator-Activated Receptor-Gama Coactivator 1 Beta
PKC	Protein Kinase C
POMC	Proopiomelanocortin
PTP-1B	Protein-tyrosine phosphatase 1B
ROS	Reactive Oxygen Species
S6K1	Ribosomal protein S6 kinase beta-1
SCD-1	Stearoyl-CoA desaturase-1
SIRT-1	Sirtuin-1
SREBP1c	Sterol Regulatory Element-Binding Protein 1c
TG	Triglycerides
TNF-α	Tumor necrosis factor alpha
TRB3	Tribbles homolog 3
VLDL	Very Low Density Lipoprotein
WAT	White Adipose Tissue
